# Empowering academic labs and scientists to test for COVID-19

**DOI:** 10.2144/btn-2020-0079

**Published:** 2020-06-25

**Authors:** James Jordan Steel, John C Sitko, Matthew G Adkins, Steven CM Hasstedt, Joseph W Rohrer, Erin A Almand

**Affiliations:** 1 Department of Biology, US Air Force Academy, Colorado Springs, CO 80840, USA; 2 10th Medical Group, US Air Force Academy, Colorado Springs, CO 80840, USA

**Keywords:** asymptomatic, COVID-19, higher education, RTPCR, SARS-CoV-2, testing

The lack of widespread COVID-19 testing and the prevalence of asymptomatic infections have been major factors in the current pandemic. Despite the improvements in clinical testing, as we move toward reopening USA, widespread surveillance testing becomes critical. Academic (nonmedical) labs can help provide such testing; the CDC-approved guidelines for COVID-19 testing require routine equipment and protocols that are commonly used in academic research labs around the country. Faculty at the authors' institution were successfully able to test asymptomatic students for COVID-19. By empowering nonmedical academic scientists with preexisting knowledge, expertise with the protocols, and access to the instruments, an additional 1.2–3.5 million COVID-19 tests could be processed each day at local universities and academic labs.

SARS-CoV-2, the causative agent for COVID-19 continues to decimate high-risk populations, cripple economies and stress an overtaxed medical system. On the front lines, medical personnel plead for increased personal protective equipment, more test kits and faster turnaround times. With this highly communicable virus, rapid testing is essential to identifying and isolating infected individuals, slowing the spread and containing the disease. Some countries, such as South Korea and Iceland, implemented widespread testing of their populations, resulting in less cases, fewer fatalities and an intact economy [1]. This inverse relationship, whereby increased COVID-19 testing leads to decreased impacts on society, means businesses and schools can reopen safely and the public health concerns remain low because the presumptive infection status of each individual is known, regardless of disease severity. Recent studies of complete populations on cruise ships and isolated aircraft carriers have shown that up to 50% of cases are asymptomatic [2–4]. These individuals do not have symptoms and therefore may not be isolating, further spreading the virus to individuals who may not be so fortunate. Despite this important, poorly understood population of asymptomatic individuals, the scarcity of reagents and the testing backlog in overworked diagnostic labs currently limits testing to symptomatic individuals. According to CDC guidelines, only hospitalized patients (Priority 1) and healthcare workers (Priority 3) are tested if asymptomatic [5]. With calls for the economy to reopen, implementation of robust testing could reduce the potential risk for a resurgent outbreak [6]. Large-scale testing programs can be (and have been) instrumental in identifying asymptomatic and presymptomatic carriers, yet this approach comes down to capacity: who will perform these tests and how will they do it? In a time of social media, crowdsourcing, citizen scientists and resource pooling, one invaluable group remarkably remains overlooked: the nonmedical academic scientist.

Early in the pandemic, efforts at universities in California [7] and Washington [8] proved the utility of research labs performing large, wide-scale testing in their communities. Academic labs have the expertise and capabilities to test for COVID-19; however, this vast resource remains untapped and left out of most public health conversations. Nonmedical academic scientists provide the scientific knowledge that allows companies to develop diagnostic kits, vaccines and therapeutics, yet a majority of them are not involved in COVID-19 testing. A recent survey of NIH-funded labs showed that nearly 40% have the capability for COVID-19 testing, but only 3% are actually involved in current testing [9]. The technique most commonly used to detect current infection is already routinely utilized across the country in labs of all sizes, and even taught to undergraduate students: quantitative reverse transcription PCR (qRT-PCR).

The technique of qRT-PCR is simple, effective and in widespread use. It is the starting point for most viral clinical diagnostics and was the first screening technique developed for SARS-CoV-2, using a nasal swab sample to screen for viral RNA from the virus. Specific primers and probes complementarily bind to the viral genome and allow highly specific identification of SARS-CoV-2 RNA, indicating the presence of the virus in the infected sample. In addition to the CDC-approved qRT-PCR protocol for detecting SARS-CoV-2 in nasal swabs, there are over 60 other USFDA Emergency Use Authorized kits. For these kits, the primers, probes and reagents are publicly available for purchase, while the diversity of manufacturers ensures there is a protocol for most labs' current setups, meaning that no additional instrumentation is required. The ease of these techniques and their widespread use in collegiate teaching and research labs means the capabilities to perform these tests exist in most college and university biology laboratories. Could this untapped capacity make a meaningful difference to the testing gap? Our research team explored the feasibility of using the expertise of PhD and MS level faculty to run the qRT-PCR CDC protocol with the currently existing infrastructure at our university.

Our team used the CDC-recommended protocol and reagents to develop a robust sampling plan for students at our university. Including the initial swabbing, the RNA extraction, qRT-PCR setup and the 75-min qRT-PCR cycling parameters, the entire protocol took under 4 h. Our sampling and protocol process had the capacity to run up to 22 samples every 90 min, the rate-limiting step being that our lab is equipped with only a single qRT-PCR thermocycler.

Due to processing restrictions based on the availability of the thermocycler, we sought to determine how common these instruments are and whether there are enough to provide a valuable resource in testing efficiency. To determine abundance of these machines, the test team used market research and conservative estimates (Table 1). The data showed a growing demand among research institutions for qRT-PCR. Market estimates show that between 9975 and 23,460 qRT-PCR or digital thermocyclers are present in research facilities in USA. Using our protocol time of 22 samples every 90 min, running for 8–10 h a day, there would be the capacity for 1.2–3.5 million tests per day. Our department was able to run these tests for roughly $8.00 per sample, not including labor costs or the equipment itself. This cost fluctuates based on protocol, with some assays costing up to $28 a sample. Despite a current opinion that there is a shortage of kits, the real shortage is the capacity of medical diagnostic labs to run the samples. Most academic labs have most of the reagents necessary to run the qRT-PCR tests. As of May 2020, Integrated DNA Technologies, the company that makes the primers and probes, has enough kits to enable approximately 33 million tests and is able to keep up with shipping out orders for SARS-CoV-2 primers and probes. By allowing academic labs to test for COVID-19, the burden of testing could be shared across universities in every state, and widespread surveillance testing could significantly increase.

**Table 1.** Estimates of the number of individuals that could be tested for SARS-CoV-2 in research institutions.

Description	Low estimate	High estimate
Estimates of the US qRT-PCR market size	$1.5 billion^†^	$1.8 billion^‡^,^§^
Research share of qRT-PCR market	38%^†^	38%^†^
Research market for qRT-PCR in USA	= $570 million	= $670 million
Market share that is equipment purchases	35%^¶^	35%^¶^
Value of qRT-PCR equipment purchased each year for research in USA	= $200 million	= $234 million
Price of qRT-PCR thermocycler	/$100,000^#^	/$50,000^#^
Number of qRT-PCR thermocyclers purchased in US research last year	= 1995	= 4681
Number of qRT-PCR thermocyclers purchased in last 5 years	= 9975	= 23,406
Hours available to run each day	x 8	x 10
Hours to run a 96-well plate	/1.5	/1.5
Patients per 96-well plate	x 22	x 22
**Number of individuals that US research institutions could test daily**	**1,170,400**	**3,432,330**

US research institutions could test between 1.2 and 3.4 million samples for SARS-CoV-2 each day if their RT-qPCR thermocyclers were fully utilized. All monetary amounts are in US dollars.

† https://www.grandviewresearch.com/industry-analysis/real-time-pcr-qpcr-digital-pcr-dpcr-market

‡ https://www.verifiedmarketresearch.com/product/global-real-time-pcr-and-digital-pcr-market-size-and-forecast-to-2025/

§ https://www.marketsandmarkets.com/Market-Reports/genomics-market-613.html

¶ https://www.alliedmarketresearch.com/polymerase-chain-reaction-technologies-market

# Based on prices listed by ThermoFisher and Bio-Rad.

**Figure 1.**
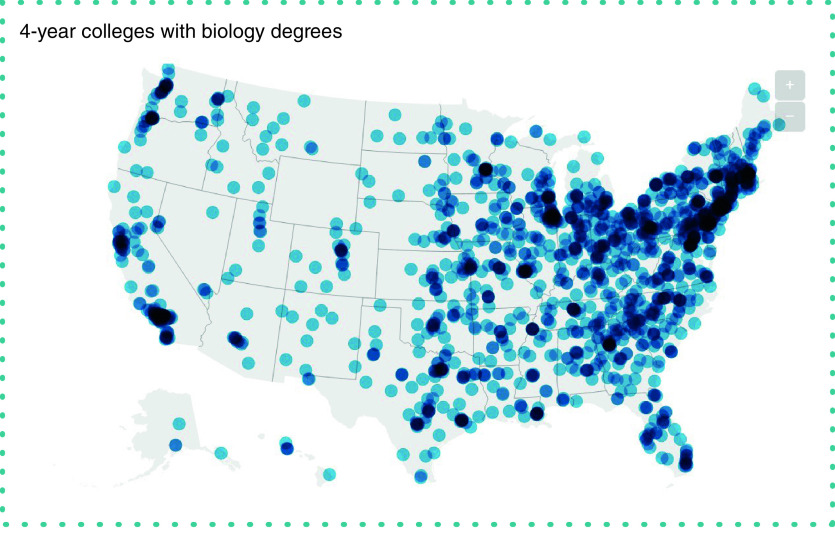
Potential nonmedical testing sites across USA. Using the College Navigator application on nces.ed.gov, institutions from across the country were selected based on the following criteria: a 4-year program and the availability of a degree in the biological and biomedical sciences. These locations were pooled and plotted using the online application Datawrapper (https://www.datawrapper.de/). Each single light-blue dot is one institution.

Based on the institutions listed with the Department of Education (Figure 1), there are currently over 1500 colleges and universities that are 4-year institutions with a program for biological and biomedical sciences. According to Higher Learning Commission accreditation guidelines for faculty members, instructors must have academic degrees relevant to what they are teaching at one level above what they teach, or a terminal degree in that field [10]. This means the individuals teaching classes at these institutions all possess at least a Master's degree in their field. These highly trained academic scientists in schools across the country could efficiently run COVID-19 tests using protocols and equipment they are already teaching undergraduate students to use.

The current barriers to using nonmedical labs for COVID-19 testing is that agencies may not treat the results as reliable because there is no Clinical Laboratory Improvement Amendments (CLIA) certification. Medical diagnostic labs must maintain CLIA certification in order to process human samples and provide medical results, whereas academic labs generally do not have this certification for their research-only endeavors. Possible ways around this problem include performing research-only studies relying on highly trained personnel and proper controls, or conducting public health surveillance studies, which are exempt under the Institutional Review Board (IRB) regulations; or CLIA certification can be acquired in connection with, and as an extension of, a previously CLIA-certified medical lab. The authors have tried both methods. Initially the COVID-19 testing was IRB-approved for research purposes only and any positive COVID-19 results were subsequently sent to the medical provider for further testing. This did not require CLIA certification and did help screen a majority of presymptomatic or subsymptomatic individuals, but unfortunately any positive results required redundant testing and close collaboration with a medical provider and lab. In order to actually report medically approved and CLIA-certified diagnostic test results, the lab must be CLIA certified. This process requires extensive paperwork, record keeping, training logs and certified equipment; however, it is feasible, especially if seeking CLIA certification as an extension of an existing CLIA-certified lab.

Another potential barrier for COVID-19 testing in an academic setting is the availability of appropriate personnel to run the tests. Despite qRT-PCR's relatively easy experimental setup, it does require trained and qualified personnel to run the assays, which would pull these scientific experts away from other productive projects. At the authors' institution, a rotation has been developed in order to help provide routine COVID-19 testing for students and staff while having minimal impact on other teaching and research requirements.

As we move to reopen our economy and brace for the next wave of this pandemic – or perhaps another pandemic altogether – we desperately need to plan for more widespread testing. We need to empower and call upon academic scientists to perform these tests. Academic and research centers have the equipment and expertise to run over 1 million COVID-19 tests per day, and this resource cannot be ignored. Nonmedical laboratories possess the competence and capacity to aid with the massive-scale testing needed to emerge from a COVID-19 lockdown. Instead of underutilizing this resource, we should harness their exceptional knowledge and scientific power to help us overcome this pandemic.
